# Confirmation Bias in the Course of Instructed Reinforcement Learning in Schizophrenia-Spectrum Disorders

**DOI:** 10.3390/brainsci12010090

**Published:** 2022-01-11

**Authors:** Dorota Frydecka, Patryk Piotrowski, Tomasz Bielawski, Edyta Pawlak, Ewa Kłosińska, Maja Krefft, Kamila Al Noaimy, Joanna Rymaszewska, Ahmed A. Moustafa, Jarosław Drapała, Błażej Misiak

**Affiliations:** 1 Department of Psychiatry, Wroclaw Medical University, Pasteur Street 10, 50-367 Wroclaw, Poland; tomaszbielawski90@gmail.com (T.B.); maja.krefft@gmail.com (M.K.); kamila.kotowicz@gmail.com (K.A.N.); joanna.rymaszewska@umw.wroc.pl (J.R.); 2 Department of Psychiatry, Division of Consultation Psychiatry and Neuroscience, Wroclaw Medical University, Pasteur Street 10, 50-367 Wroclaw, Poland; tryku@yahoo.com (P.P.); mblazej@interia.eu (B.M.); 3 Department of Experimental Therapy, Institute of Immunology and Experimental Therapy, Polish Academy of Sciences, Weigel Street 12, 53-114 Wroclaw, Poland; edyta.pawlak@hirszfeld.pl; 4 Day-Care Psychiatric Unit, University Clinical Hospital, Pasteur Street 10, 50-367 Wroclaw, Poland; ewa.klosinska@gmail.com; 5 School of Psychology, Marcs Institute for Brain and Behaviour, Western Sydney University, Locked Bag 1797, Penrith, NSW 2751, Australia; ahmedhalimo@gmail.com; 6 Department of Human Anatomy and Physiology, Faculty of Health Sciences, University of Johannesburg, Johannesburg 2006, South Africa; 7 Department of Computer Science and Systems Engineering, Faculty of Information and Communication Technology, Wroclaw University of Science and Technology, Wybrzeze Wyspianskiego Street 27, 50-370 Wroclaw, Poland; jaroslaw.drapala@gmail.com

**Keywords:** schizophrenia, instructed reinforcement learning, confirmation bias

## Abstract

A large body of research attributes learning deficits in schizophrenia (SZ) to the systems involved in value representation (prefrontal cortex, PFC) and reinforcement learning (basal ganglia, BG) as well as to the compromised connectivity of these regions. In this study, we employed learning tasks hypothesized to probe the function and interaction of the PFC and BG in patients with SZ-spectrum disorders in comparison to healthy control (HC) subjects. In the Instructed Probabilistic Selection task (IPST), participants received false instruction about one of the stimuli used in the course of probabilistic learning which creates confirmation bias, whereby the instructed stimulus is overvalued in comparison to its real experienced value. The IPST was administered to 102 patients with SZ and 120 HC subjects. We have shown that SZ patients and HC subjects were equally influenced by false instruction in reinforcement learning (RL) probabilistic task (IPST) (*p*-value = 0.441); however, HC subjects had significantly higher learning rates associated with the process of overcoming cognitive bias in comparison to SZ patients (*p*-value = 0.018). The behavioral results of our study could be hypothesized to provide further evidence for impairments in the SZ-BG circuitry; however, this should be verified by neurofunctional imaging studies.

## 1. Introduction

Humans learn how to behave both through rules and instructions as well as through environmental daily experiences. There are various dual-process models describing separable decision-making systems that contribute to rule-based/instructed choices versus those based on experience/procedural learning [1]. A substantial body of research suggests that the prefrontal cortex (PFC) is active during rule-based learning [2] and plays a role in rule-governance effects [3] and rule-retrieval [4], whereas integration of information over trials elicits basal ganglia (BG) activity, mainly in the striatum [3]. Systems dependent on the PFC encode task instructions and rapidly update representations based on single outcomes for flexible control of choices [5], while systems based on the BG integrate reinforcement contingencies slowly by trial and error to support maximally adaptive responses to current stimuli [6,7]. However, there are still a lot of conflicting reports about how these learning systems cooperate or compete for control of behavior in order to employ the best strategy to adapt to changing environmental conditions.

The interaction between different systems in the brain is an especially important issue in schizophrenia (SZ), since one of the main features associated with the disorder is disintegration of conscious experience. There has been a growing body of research showing that different psychopathological symptoms may result from dysfunctional connectivity between distributed brain areas [8]. SZ has been associated with reduced functional connectivity across fronto-temporal networks supporting verbal encoding [9], hippocampal-prefrontal and prefrontal-thalamic networks during working-memory tasks [10,11], occipito-temporal networks supporting semantic processing [12], and dysfunctional integration between brain regions involved in reinforcement learning [13,14]. The recent meta-analysis of studies on resting-state functional connectivity among at-risk-of-psychosis individuals supports the hypothesis that large-scale network dysfunctions represent a core neural deficit underlying psychosis development [15]. For a long time, most of the studies discussed potential mechanisms of impairment in schizophrenia with respect to separate neural systems that could independently contribute to cognitive deficits in goal-directed behavior. However, in recent years, studies have looked into impairments in several systems with the focus on dysfunctional interactions between them in order to gain deeper understanding of the neural basis of cognitive impairments in SZ [16].

The Instructed Probabilistic Selection Task (IPST) is a task variant of a widely studied probabilistic reinforcement learning (RL) task that has repeatedly been shown to be associated with dopaminergic effects on learning [6] and is consistent with PET [17] and fMRI [18,19] studies showing BG activation using this task. In the IPST, participants receive false instruction about the higher value of one of the stimuli used in the probabilistic reinforcement learning task. The IPST is hypothesized to interrogate the interaction of the PFC and BG and produce confirmation bias, whereby the instructed stimulus is overvalued in comparison to its real experienced value [20,21,22,23]. Such instructions can have detrimental effect when they are inaccurate, whereby data that are consistent with that information are sought or valued over disconfirming data, which are filtered or neglected. RL depends on dopaminergic prediction errors, through which BG, mainly the striatum, can learn the value of the action [24,25]. Explicit instructions are proposed to bias striatal learning through the influence of PFC [23,26], which enables rules and instructions to influence goal-directed behavior [27]. Connectivity analyses further support the role of PFC, reporting increased functional connectivity between PFC an BG regions during instructed/prior knowledge conditions [28,29].

There is a body of research in patients with SZ showing deficits in learning tasks in which explicit hypotheses are tested and evaluated on a trial-to-trial basis, typically interpreted as the PFC dysfunction [30,31]. Moreover, there are also reports indicating deficits in implicit forms of RL, mainly in tasks relying primarily on positive and negative feedback-driven procedural learning mechanisms, typically interpreted as the BG dysfunction attributed to alterations of phasic striatal dopamine signals [30,32,33]. It is still not clear how these two systems interact and contribute to cognitive dysfunctions observed at a behavioral level. Thus, the aim of our study was to assess confirmation bias in the course of instructed RL in patients with SZ in comparison with healthy control (HC) participants by employing the Instructed Probabilistic Selection Task (IPST).

This task has been hypothesized to probe the function as well as interaction of PFC and BG [22], and several neurocomputational models have been designed in an attempt to distinguish between the mechanisms governing instructed behavior. The best-fitting model supports the neural model, suggesting the existence of a confirmation bias in which PFC influences BG by amplifying outcomes consistent with the instruction and diminishing inconsistent outcomes [20]. Thus far, to the best of our knowledge, there is only one study using this task to compare performance of patients with SZ with HC subjects; however, both studied groups were very small (48 patients with SZ and 38 HC participants) [22]. The authors of this study showed reduced confirmation bias among patients in comparison to HC participants, which has been attributed by the authors to reduced prefrontal-striatal communication that led to a paradoxical improvement in patients’ performance on the task. We aimed at using IPST on a bigger sample and further analyze in detail not only the initial influence of the instruction on the probabilistic RL, but also the process of overcoming false information due to receiving feedback that is conflicting with the initial instruction.

We hypothesized that patients with SZ will perform worse in the acquisition of reinforcement contingencies, and they will be less prone to confirmation bias that is ameliorated by feedback at a slower pace in comparison to HC participants. The acquisition of contingencies will be assessed by the accuracy of responses in the training and testing phase of the task. The confirmation bias will be assessed by comparing of the accuracy of responses on the trials with the false instruction in comparison to the trials with the same contingencies but without a false instruction.

## 2. Materials and Methods

### 2.1. Participants

In our study, we included 102 patients with schizophrenia-spectrum disorders: 18% with schizoaffective disorder, 64% with paranoid schizophrenia and 18% with first-episode psychosis (57 males/45 females, aged 39.19 ± 13.81 years) and 120 healthy control (HC) participants (45 males/75 females, aged 36.80 ± 18.34 years). A diagnosis of SZ was based on the DSM-IV and ICD-10 criteria validated using the Operational Criteria for Psychotic Illness (OPCRIT) checklist [34]. The recruitment of patients with SZ and HC participants took place within the years 2015–2019. Patients with SZ were recruited from inpatient and outpatient units in the Lower Silesian area, Wroclaw, Poland. The HC participants were recruited from the general population via advertisement and via word of mouth. There were the following exclusion criteria for the participants: general brain disorder, intellectual disability, severe physical health impairments, and comorbid drug- and/or alcohol-use disorder (except nicotine dependence). HC participants had no personal or family history of mental illness. All participants of the study were of Caucasian origin. There were 85% of patients recruited from inpatient settings and 15% of patients recruited from outpatient settings. In addition to antipsychotic treatment, there were 22 (21%) patients treated additionally with antidepressants, 15 (15%) patients treated additionally with anticonvulsants, 3 (3%) patients treated additionally with lithium, and 3 (3%) patients were currently receiving benzodiazepines. The study was conducted according to the guidelines of the Declaration of Helsinki and approved by the Ethics Committee of Wroclaw Medical University (KB-59/2015, date of approval 5 March 2015). All participants gave written informed consent. The participants completed general neuropsychological assessment. Overall symptom severity in patients was assessed by board-certified clinicians.

### 2.2. Measures

#### 2.2.1. Clinical Assessment

Clinical manifestation was assessed using the Brief Psychiatric Rating Scale (BPRS) (Overall and Gorman, 1962), the Scales for the Assessment of Negative Symptoms (SANS) and Positive Symptoms (SAPS) [35], the Positive and Negative Syndrome Scale (PANSS) [36], the Montgomery-Asberg Depression Rating Scale (MADRS) [37], and the Hamilton Depression Rating Scale (HDRS) [38]. General functioning was recorded using the Global Assessment of Functioning scale (GAF) (American Psychiatric Association, 1994). The dosage of antipsychotics was expressed as chlorpromazine equivalents (CPZeq) in mg/day [39].

#### 2.2.2. General Neuropsychological Assessment

Participants were assessed with respect to cognitive performance on the Repeatable Battery for the Assessment of Neuropsychological Status (RBANS) [40]. The RBANS is a brief, neuropsychological screening measure. It consists of twelve subtests that can be combined into five domains: immediate memory (list learning and story memory), visuospatial/constructional (figure copy and line orientation), language (picture naming and semantic fluency), attention (digit span and coding), and delayed memory (list recall, list recognition, story recall, and figure recall).

#### 2.2.3. The Instructed Probabilistic Selection Task (IPST)

**Figure 1:**
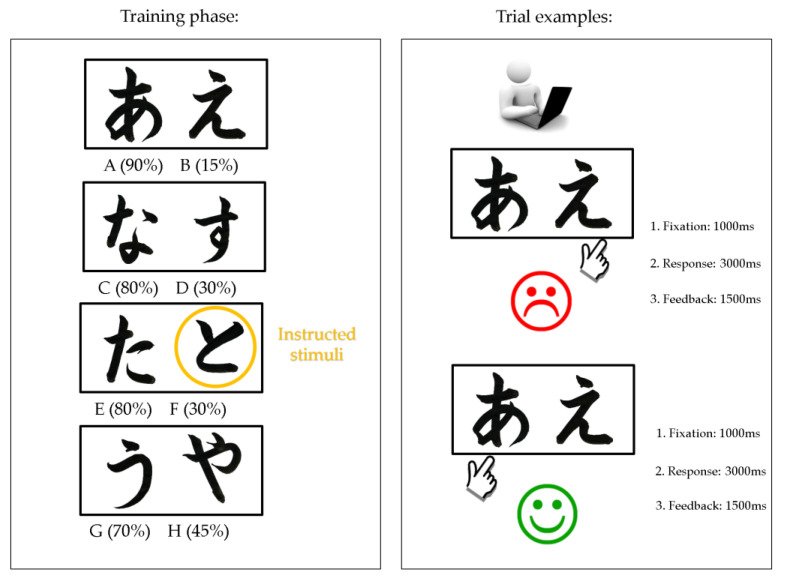
Probabilistic contingencies during training phase of IPST AB (90%/15%), CD (80%/30%), EF (80%/30%), and GH (70%/45%) conditions and trial examples with negative and positive feedback.

To assess the effect of instruction on the process of RL, we used a computerized version of the Instructed Probabilistic Selection Task (IPST) [20,22]. The structure of the task is shown in Figure 1. The task has training and testing phases. There are four stimulus pairs used in the task (AB, CD, EF, GH). Each stimulus is represented by a unique Japanese Hiragana character to minimize explicit verbal encoding. During the training phase, at each trial participants are required to choose between stimulus pairs. Feedback follows the choice to indicate whether it was correct or incorrect, but this feedback is probabilistic, with one stimulus in each training pair being more likely to be rewarded (AB 90%/15%, CD 80%/30%, EF 80%/30%, GH 70%/45%). For example, in AB trials, a choice of stimulus A leads to correct (positive) feedback in 90% of AB trials, whereas a B choice leads to incorrect (negative) feedback in these trials (and vice versa for the 15% of trials). It should be noted that learning to choose A over B could be accomplished either by learning that choosing A leads to positive feedback, or that choosing B leads to negative feedback (or both). Participants were instructed that in each stimulus pair that one stimulus is better than the other, although there was no absolutely correct answer. Additionally, participants received inaccurate information that the value of stimulus F was the highest. Before completing the task, subjects read the task instruction that appeared on the screen:

“Two black symbols will appear simultaneously on the computer screen. One symbol will be ‘correct’ and the other will be ‘incorrect’, but at first you will not know which is which. Try to guess the correct figure as quickly and accurately as possible. There is no absolute right answer, but some symbols will have a higher chance of being correct than others. Try to pick the symbol you find to have the highest chance of being correct. This symbol has the highest probability of being correct [symbol F is being shown]. You will have to figure out which of the other symbols you should select by trying them out. Now you will be tested on these instructions to make sure you have understood them fully”.

Participants were tested on comprehension of this instruction before the beginning of the task. They were asked (1) how many stimuli would appear on the screen at a time, (2) how to select the left and right stimuli, and (3) shown all of the stimuli and asked to select the stimulus they were told would have the highest chance of being correct. Incorrect answers on any questions restarted the instructions and subsequent test. They were reinstructed until performance of the comprehension test was satisfactory. Training blocks consisted of 40 trials. The training phase was terminated when participants either achieved a criterion as defined by 70% correct in the AB condition, 65% correct in the CD condition, and 60% correct in the GH condition, or after 160 trials were completed. This criterion was intended to prevent overlearning of the contingencies prior to the testing phase [33]. Each participant was given a randomly chosen version of a stimulus pair associated with reward probability since it has been shown that stimulus discriminability may bias value-based probabilistic learning [41]. Following the training phase, participants completed the testing phase, involving combinations of paired stimuli presented in random order, with each combination repeating four times. Participants were informed that they should pick the symbol they felt was correct more often on the basis of what they had learned during training. The test phase started with reading the following instructions:

“It is time to test what you have learned. During this set of trials, you will not receive feedback (‘correct’ or ‘incorrect’) to your responses. If you see a new combination of symbols in the test, please choose the symbol that ‘feels’ more correct based on what you learned during the training sessions”.

### 2.3. Statistics

The comparison of continuous variables was performed using the analysis of variance (ANOVA) in case of normal distribution (the Kolmogorov–Smirnov test *p*-value greater than 0.05) and homogeneity of variance (Levene’s test of homogeneity *p*-value greater than 0.05) of the given variable. For the comparison of continuous variables that did not meet those criteria, we used either the Mann–Whitney U test or the Kruskal–Wallis test depending on the number of categories. For the comparison of categorical variables, the χ^2^ test was used. A two-way ANOVA was performed to test the effects of group (SZ vs. HC participants) and conditions as well as the interaction between them on the training phase performance in CD and EF condition. Post-hoc comparisons were performed using the Tukey test. In order to assess the performance on the first 10 trials until the last 10 trials of the EF condition, we performed repeated-measures ANOVA with the Tukey post-hoc comparisons between patients with SZ and HC participants. At test phase, performance between SZ patients and HC subjects on the DF pair was compared, which pairs the instructed stimulus F against uninstructed stimulus D, both of which had identical reward probability during the training phase. Moreover, the effect of instruction at test phase was assessed with a two-way ANOVA with factors of groups and the Avoid-D/Avoid-F measures. The comparison between performance on pairs AD and DE (Avoid-D) versus performance on the pairs AF and CF (Avoid-F) was chosen, because stimuli D and F had the same reward probability during the training phase and thus the participants should perform equally well on both measures. However, if false instruction biased the learned reward value of stimulus F, avoiding stimulus F should be at a lower rate in comparison with avoiding stimulus D. In correlational analysis of continuous variables, we used the Pearson’s and Spearman’s correlation coefficients depending on the normality of data distribution according to the Kolmogorov–Smirnov test. We performed correlation analyses to assess relationships between performance on IPST and clinical-symptom ratings (the SANS, SAPS, PANSS, MADRS, BPRS, and HDRS scores), general functioning (GAF), CPZeq, and neurocognitive functioning (RBANS). We used non-parametric analyses for the following variables: accuracy during GH trials, RBANS, PANSS (positive, negative, general symptoms),and SAPS and SANS and illness duration. Bonferroni correction was used due for multiple testing in correlational analyses. Due to 18 clinical-cognitive measures, the significant *p*-value is less than 0.0028. All tests were two-tailed with the level of significance set at *p*-value less than 0.05. Statistical analysis was performed using the Statistical Package for Social Sciences, version 20 (SPSS Inc., Chicago, IL, USA).

## 3. Results

General demographic and clinical characteristics of the sample with respect to SZ and HC groups are presented in Table 1.

### 3.1. Reinforcement Learning in Instructed Probabilistic Task (IPST)

**Figure 2:**
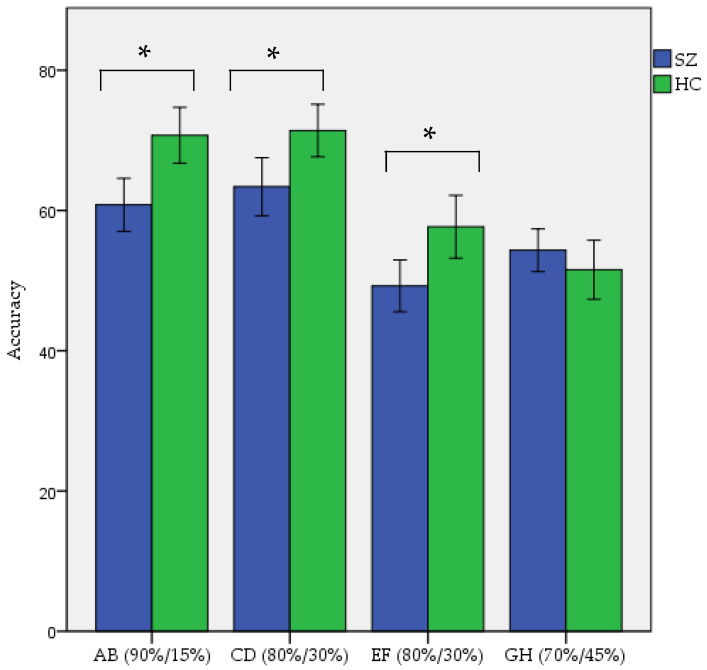
Comparison of acquisition of probabilistic contingencies during IPST for AB (90%/15%), CD (80%/30%), EF (80%/30%), and GH (70%/45%) conditions between schizophrenia (SZ) patients and healthy control (HC) participants. Green color refers to HC participants and blue color refers to the patients with SZ, * refers to statistically significant differences.

One-way ANOVA assessing the difference in accuracy between patients with SZ and HC participants on the summary measure created by averaging the proportion of correct responses from all four conditions of each stimulus pair (AB, CD, EF, and GH) during the training phase of IPST showed better overall performance of HC participants (61.41 ± 11.12) in comparison to patients with SZ (56.94 ± 11.82) (F = 8.41, *p*-value = 0.004). Assessing differences in accuracy between patients with SZ and HC participants in each stimulus pair showed significantly better performance of HC participants compared to patients with SZ on the AB (90%/15%) condition (F = 9,10, *p*-value = 0.003), CD (80%/30%) condition (F = 5.53, *p*-value = 0.020), EF (80%/30%) condition (F = 6.62, *p*-value = 0.011), and no statistically differences in performance on the GH (70%/45%) condition (Z = −1.35, *p*-value = 0.178). Proportions of correct responses given by participants during the training phase of the IPST with respect to each condition (AB, CD, EF, and GH) are shown in Figure 2. A two-way ANOVA, with factors of groups and only uninstructed reinforcement contingency conditions that were normally distributed and had homogenous variances (AB and CD), showed statistically significant differences between patients with SZ and HC participants in the AB condition (*p*-value = 0.035) and only trend-level significant differences in the CD condition (*p*-value = 0.089).

Correlational analyses between averaged performance measure on the IPST across all reward conditions (AB, CD, EF, and GH) in the whole group (patients with SZ and HC subjects) showed association with age (r = −0.18, *p*-value = 0.007), and the total RBANSS score (r = 0.22, *p*-value = 0.001), in particular with the measures of learning (r = 0.22, *p*-value = 0.001), immediate memory (rho = 0.21, *p*-value = 0.002), attention (r = 0.18, *p*-value = 0.008), and delayed memory (r = 0.18, *p*-value = 0.009). Among patients with SZ, we found a significant association of averaged performance measure on the IPST with illness duration (rho = −0.232, *p*-value = 0.032). No significant associations between performance during acquisition phase on the IPST and clinical measures (SAPS, SANS, BPRS, MADRS, and BDI), general functioning (GAF), CPZeq or age of onset were observed (*p*-value > 0.05). However, we found significant negative associations between the RBANS total score and the PANSS negative symptoms (rho = −0.33 *p*-value = 0.002), the PANSS total score (r = −0.30, *p*-value = 0.004), the BPRS score (r = −0.25, *p*-value = 0.030), and the CPZeq (r = −0.23, *p* = 0.027), as well as a significant positive association between the RBANS total score and the GAF (r = 0.29, *p* = 0.014). There were significant differences with respect to sex and educational level in our samples, so we assessed the associations sex and educational level with the average performance during the IPST in order to rule out the confounding effect of these variables. In fact, we did not find significant differences in the performance in the training phase with respect to sex or with educational level among SZ patients (F = 0.89, *p*-value = 0.349, χ^2^ = 4.03, *p* = value = 0.258, respectively) and among HC subjects (F = 0.33, *p*-value = 0.568, χ^2^ = 0.48, *p* = value = 0.786, respectively). After application of the Bonferroni correction, significant associations remained between averaged performance measure on the IPST across all reward conditions in the whole group with the total RBANSS score (rho = 0.22, *p*-value = 0.001) and between the RBANS total score and the PANSS negative symptoms (rho = −0.33 *p*-value = 0.002).

### 3.2. The Effect of Instruction in the Instructed Probabilistic Task (IPST)

We assessed the effect of instruction on probabilistic learning both in the training phase and testing phase of the IPST. The effect of instruction was measured during the training phase by comparing performance with respect to two conditions with the same reinforcement contingencies (80%/30%); however, one was an uninstructed (CD) while the other was an instructed (EF) condition.

**Figure 3:**
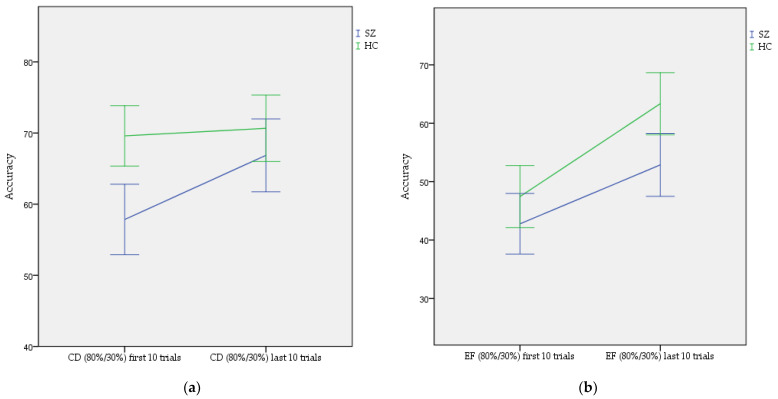
Comparison of performance during the training phase of IPST among schizophrenia (SZ) patients and healthy control (HC) participants: (**a**) first and last 10 trials of uninstructed CD (80%/30%) condition; (**b**) first and last 10 trials of instructed EF (80%/30%) condition. Green color refers to HC participants and blue color refers to the patients with SZ.

First, in order to assess the initial impact of instruction we tested the difference between the first 10 trials of the CD condition and first 10 trials of the EF condition in patients with SZ and HC participants. A two-way ANOVA with factors of groups and the first 10 trials of both the CD and EF conditions showed statistically significant main effects of group (F = 10.27, *p*-value = 0.001) and condition (F = 55.256, *p*-value < 0.001), but not a significant effect of the interaction between these variables (F = 1.72, *p*-value = 0.190). Post-hoc analysis showed statistically significant differences between first 10 trials of the CD condition and first 10 trials of the EF condition among patients with SZ (57.84 ± 25.24 and 42.77.84 ± 26.43, respectively, *p*-value < 0.001) and HC participants (68.96 ± 22.38 and 47.92.96 ± 27.83, respectively, *p*-value < 0.001), and no significant differences in the first 10 trials of the EF condition between patients with SZ and HC participants (42.77.84 ± 26.43 and 47.92.96 ± 27.83, respectively, *p*-value = 0.441). This means that false F instruction being the best stimulus influenced acquisition of correct contingencies during the training phase of the IPST both in the group of patients with SZ as well as among HC participants. The results are shown in Figure 3.

**Figure 4:**
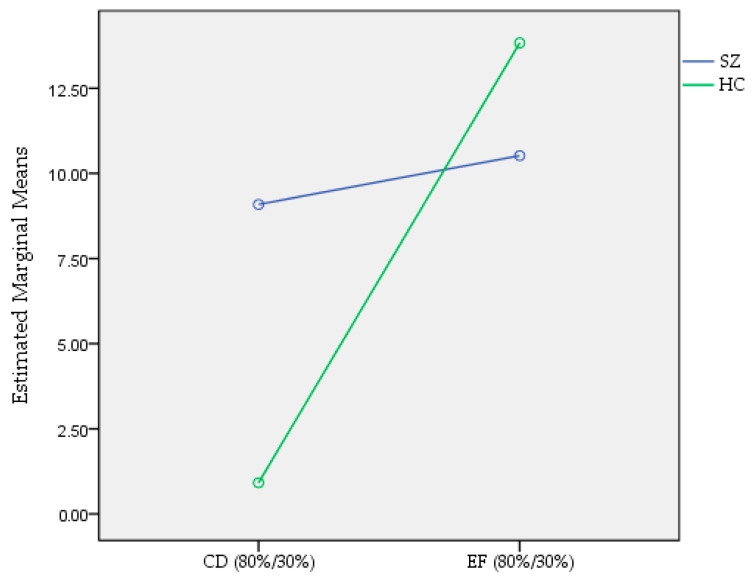
The effect of interaction of group (patients with SZ and HC subjects) and the difference between the first 10 and last 10 trials of the CD (80%/30%) and EF (80%/30%) conditions of the training phase of the IPST. Green color refers to HC participants and blue color refers to patients with SZ.

Second, in order to assess the process of overcoming false instruction, we compared performance with time in the EF condition among patients with SZ and HC participants. Repeated-measures ANOVA showed a significant main effect of time (F = 26.12, *p*-value < 0.001) with medium effect size (partial eta squared = 0.11), but no effect of the interaction between group and time (F = 0.64, *p*-value = 0.426). Post-hoc tests showed significant differences between patients with SZ and HC participants (F = 5.66, *p*-value = 0.018). Additionally, we compared differences between the first 10 trials and last 10 trials of the CD and EF condition in patients with SZ and HC participants. A two-way ANOVA with factors of groups and the difference between the first 10 and last 10 trials of the CD condition and between the first 10 and last 10 trials of the EF condition showed statistically significant main effects of group (F = 31.57, *p*-value = 0.022), no statistically significant main effect of the difference between the first 10 and last 10 trials of CD and EF conditions (F = 0.48, *p*-value < 0.001), and a statistically significant effect of the interaction of group and the difference between the first 10 and last 10 trials of the CD and EF conditions (F = 4.04, *p*-value = 0.045) (Figure 4). Post-hoc analyses showed that there was a significant difference in learning speed between the CD and EF conditions in HC participants (0.91 ± 27.07 and 13.03 ± 34.79, respectively, *p*-value = 0.008), while there was no significant difference in patients with SZ (9.02 ± 27.70 and 10.10 ± 34.74, respectively, *p*-value = 0.995).

Group differences in the test-phase performance of the IPST using measures for choosing each stimulus in the old and novel pairings (i.e., AD, BD, GD, or AF, CF, and GF) did not show any significant differences between patients with SZ and HC participants (*p*-value > 0.05). In addition, we used both measures described in the literature to assess the effect of instruction on the test-phase performance [22,42]. In the first analysis, we compared performance on the Avoid-D (AD, DE) versus performance on the Avoid-F (AF, CF). In both cases, the target stimulus should not be selected, as it has been paired with stimuli that during the training phase had a higher probability of reward. Stimuli D and F have identical reward probability during training, so the participants should perform equally well on both measures. However, if instruction biased the learned reward value of stimulus F, avoiding stimulus D should be at a lower rate in comparison with avoiding stimulus D. A two-way ANOVA with factors of groups and the Avoid-D/Avoid-F measures did not show significant effects of group (F = 0.05, *p*-value = 0.822), Avoid-D/Avoid-F testing measure (F = 1.89, *p*-value = 0.170), and the group × Avoid-D/Avoid-F testing measure interaction (F = 0.59, *p*-value = 0.443). The second analysis of instructed learning examined performance on DF trials in order to directly compare the relative subjective value of the two stimuli. A greater effect of instruction on learning should be associated with an increased tendency to choose stimulus D over stimulus F. We did not find a significant difference in choosing stimulus F in the DF pairs between patients with SZ and HC participants (F = 0.11, *p*-value = 0.745).

## 4. Discussion

In our study, we employed the RL task with instruction (IPST) that is hypothesized to probe the function and interaction of the PFC and BG [20,21]. Our aim was to assess striatal functions (probabilistic learning accuracy) and fronto-striatal communication (influence of the instruction on learned contingencies, known as confirmation bias) in patients with SZ and HC participants. The overall results of the study suggest that patients with SZ perform poorer in comparison to HC participants in the acquisition of reinforcement contingencies. This is in line with other studies showing impaired probabilistic RL among patients with SZ either attributed to impaired reward-driven learning [33,43] or deficits in learning from both positive and negative feedback [44,45]. SZ is known to be associated with increased stochasticity in dopamine levels [46] that may result in a slower integration of reward statistics over time and thus an impaired rate of learning [45]. Although patients with SZ performed worse overall in comparison with HC participants, these effects were most apparent in the most deterministic learning condition (AB, 90%/15% reward probability). A similar effect was observed in other studies [22,43] and has been explained by partially dissociable neural substrates in deterministic, as compared to probabilistic, discriminations. The discriminations that are nearly deterministic may rely on rule [4] or value representations [47] in the PFC in addition to BG function. Indeed, studies on PFC damage indicate association with impairments in maximizing reward in discriminations with low, rather than high, stochasticity [48].

**Figure 5:**
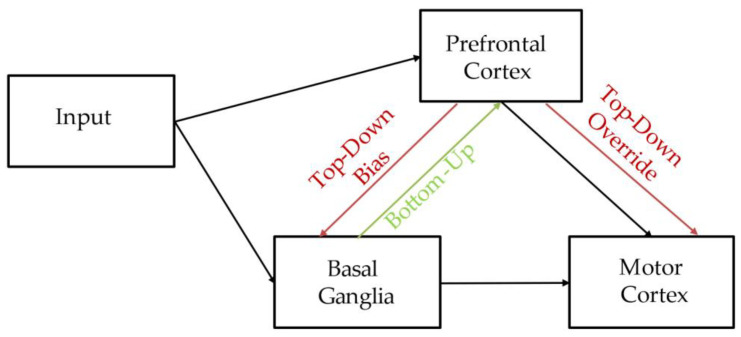
Diagram depicting neural network accounts of instructional control over reinforcement learning. Red lines indicate projections with differing computational roles. Instruction representations from PFC via top-down projections either directly bias the BG valuation, selection, and learning (bias model), or override BG learning of probabilities through the competition at motor cortex (override model) (red line). Bottom-up connections between BG and PFC enable updating of the cortical value of falsely instructed stimuli (green line).

Moreover, we showed that both patients with SZ and HC participants were initially affected by false instruction to the similar extent. Our results are consistent with research showing that both groups are similarly affected by false instruction at the beginning of the training phase of the task [22]. In general, there are two main mechanisms proposed to describe PFC and BG interactions supported by behavioral genetics and neuroimaging studies. The first model suggests that instructions bias striatal mechanisms via input from PFC that amplifies positive prediction errors and diminishes negative ones (bias model) [21,23,26,49]. The second model posits that prefrontal instruction representations override the striatum to control behavior (override model) [28,50]. These models are presented in a diagram (top-down projections are marked in red) [21] (Figure 5). Although a complete understanding of the neural mechanisms by which instructions exert control of actions is lacking, and there are conflicting results as to whether PFC and BG cooperate or compete, fronto-striatal coordination has repeatedly been implicated to be the source of confirmation bias [20].

Our results show that, among patients with SZ and HC participants, initial bias toward the inaccurately recommended stimulus was gradually shifted as participants received continued negative feedback; however, patients with SZ were overcoming confirmation bias at a slower pace in comparison with HC participants. Interestingly, we additionally showed that HC participants have higher learning speed under instructed (EF) in comparison with uninstructed (CD) conditions, while learning speed among patients with SZ in both conditions is similar. This suggests that the mismatch between instruction (encoded by PFC) and real value of the instructed stimuli (encoded by BG) influences the speed of overcoming confirmation bias. Although there are numerous studies explaining the occurrence of confirmation bias due to top-down projections from PFC, as described above, the process of overcoming false instruction has received less attention. Here, we propose that poorer performance among patients with SZ in comparison with HC participants might be due to worse bottom-up integration in PFC–BG communication that might be crucial in overcoming the influence of false instructions. We propose the modification of a diagram used to explain instructional control of PFC over BG [21] to include bottom-up projections (marked in green) (Figure 5). Indeed, there are studies showing that patients with SZ are impaired at transferring implicit knowledge on the explicit level. There are studies reporting that patients with SZ show relatively preserved learning rates in several learning procedures on the implicit level [51]; however, they have difficulty when requested to report their knowledge explicitly [52,53,54]. Moreover, a similar pattern of performance is observed among patients with temporo-hippocampal and diencephalic lesions—patients are able to increase their performance in the implicit tasks but fail to establish an explicit knowledge of category cues [55,56]. Interestingly, patients with SZ show intact performance in skill-learning tasks that is impaired in BG diseases [57,58]. An interesting perspective for future studies could be to evaluate whether a cognitive remediation intervention, which represents an evidence-based intervention to treat cognitive deficits [59], is capable of reducing or even normalizing this specific cognitive bias in SZ patients.

There are several limitations of our study. Firstly, SZ is a highly diverse disorder with different symptom dimensions and a variety of cognitive deficits. We found no significant associations between the performance on the IPST task and clinical measures or general functioning of the patients; however, behavioral results obtained in our study depend on the sampling methods, such as the percentage of patients with first-episode psychosis or schizoaffective disorder, the predominant symptomatology, or currently prescribed treatment. This variability of SZ has been previously argued for the differences in the results obtained by different authors on RL tasks [33,43,60]. Additionally, although we did not find any antipsychotic medication effect on task performance in patients with SZ, we are unable to rule out its confounding the effect of the overall clinical severity of illness on cognition. Future studies should assess behavioral effects in medication-naïve patients or among patients with randomized antipsychotic medication type and dosage in order to resolve this issue. In addition to antipsychotic treatment, the patients in our study were also prescribed non-antipsychotic drugs (antidepressants, anticonvulsants, lithium, and benzodiazepines), and the effect of these medication types has not been yet studied in detail with respect to RL in SZ. It must be also noted that, in our study, we provide only behavioral results of an instructed probabilistic RL task and thus further neurofunctional imaging studies must verify whether the obtained results indeed are associated with the impaired connectivity of PFC and BG in SZ. Moreover, we failed to show differences in confirmation bias in the test phase of the task both in patients with SZ and among HC participants. This effect might be attributed to the use of Hiragana characters in the task, which results in difficulty in conscious encoding of stimuli [33]. Participants were thus able to update their knowledge about the contingencies of the stimuli on the implicit level during the training phase; however, they had to explicitly assess their values in the test phase. In future studies, images of common objects could be used as stimuli to verify this hypothesis.

## 5. Conclusions

In conclusion, patients with SZ and HC participants were equally influenced by false instruction in an RL probabilistic task (IPST); however, HC participants had significantly higher learning rates associated with the process of overcoming cognitive bias compared to patients with SZ. Our results could be interpreted as a sign of impairment in the top-down and bottom-up connectedness between PFC and BG in SZ; however, this should be further verified with neuroimaging studies.

## Figures and Tables

**Table 1:** General demographic and clinical characteristic of schizophrenia (SZ) patients and healthy control (HC) participants.

Category	Variable	SZ	HC	*p*-Value
Demographic information	Age (years)	38.19 ± 13.81	36.80 ± 13.34	0.528 ^1^
	Sex (M/F)	5712/45	45/75	0.005
	Educational level (%)	-	-	<0.001
	Primary	19.8%	11.7%	
	Vocational	9.9%	0.0%	
	Secondary	44%	48.7%	
	Higher	26.4%	39.6%	
Neurocognition (mean ± SD)	RBANS—immediate memory	38.78 ± 11.20	50.66 ± 7.08	<0.001 ^2^
	RBANS—visuospatial and constructional	33.06 ± 6.51	37.24 ± 3.27	<0.001 ^2^
	RBANS—language	28.55 ± 6.73	34.61 ± 6.71	<0.001 ^1^
	RBANS—attention	43.99 ± 13.40	63.66 ± 14.10	<0.001 ^1^
	RBANS—delayed memory	42.38 ± 11.57	54.21 ± 5.86	<0.001 ^2^
	RBANS—total score	187.25 ± 39.88	240.67 ± 29.17	<0.001 ^2^
Clinical ratings (mean ± SD)	Age of onset	24.82 ± 7.42	-	-
	Illness duration	12.13 ± 10.56	-	-
	BPRS	40.02 ± 10.48	-	-
	PANSS—positive symptoms	13.34 ± 4.71	-	-
	PANSS—negative symptoms	21.04 ± 9.60	-	-
	PANSS—general symptoms	29.35 ± 7.97	-	-
	SANS	33.46 ± 22.84	-	-
	SAPS	20.09 ± 20.19	-	-
	MADRS	8.33 ± 9.00	-	-
	GAF	47.08 ± 20.54	-	-
Antipsychotic medication (mean ± SD)	CPZeq	501.98 ± 340.52	-	-

^1^ parametric test, ^2^ non-parametric test, abbreviations: RBANS—Repeatable Battery for the Assessment of Neuropsychological Status; BPRS—Brief Psychiatric Rating Scale; PANSS—Positive and Negative Syndrome Scale; SANS—Scale for the Assessment of Negative Symptoms; SAPS—Scale for the Assessment of Positive Symptoms; MADRS—Montgomery–Asberg Depression Rating Scale; GAF—Global Assessment of Functioning scale; CPZeq—chlorpromazine equivalent, SD—Standard Deviation, SZ—schizophrenia-spectrum patients; HC—healthy control participants.

## Data Availability

The data presented in this study are available on request from the corresponding author.
